# Dermoscopy of Scalp Melanoma: Report of Three Cases

**DOI:** 10.3390/cancers2031597

**Published:** 2010-08-18

**Authors:** Fernanda Torres, Gabriella Fabbrocini, Sergio Henrique Hirata, Sergio Yamada, Valerio De Vita, Maria Carmela Annunziata, Antonella Tosti

**Affiliations:** 1 Institute of Dermatology of Rio de Janeiro (IDERJ), Rio de Janeiro, Brazil; E-Mail: fernandantorres@hotmail.com; 2 Department of Systematic Pathology, Division of Dermatology, University of Naples Federico II, Naples, Italy; E-Mail: gafabbro@unina.it; 3 Department of Dermatology, Federal University of São Paulo (UNIFESP), São Paulo, Brazil; E-Mails: yamada.derm@epm.br (S.Y.); serhir@hotmail.com (S.H.H.); 4 Department of Internal Medicine, Geriatrics and Nephrology, Division of Dermatology, University of Bologna, Bologna, Italy; E-Mail: tosti@hotmail.com

**Keywords:** scalp melanoma, dermatoscopy

## Abstract

Scalp melanoma is rare and often late-discovered because of its unusual position. As a consequence, its prognosis is poorer than melanoma on other body sites and only few clinical reports about its dermoscopic pattern have been published. In this paper, we report three clinical cases of scalp melanoma with photographic documentation and dermoscopic images, in order to improve the early detection of scalp melanoma.

## 1. Introduction

The incidence of cutaneous malignant melanoma has increased steadily over the past 30 years in all Western countries and this has been accompanied by a similar but less marked increase in mortality [1,2,3,4]. The prognosis of malignant melanoma is related to a number of factors including the Breslow thickness, clinical subtypes, ulceration and tumor site. In particular, scalp melanoma—defined as a melanoma arising on the usually hair-bearing area of the head—has a significantly worse prognosis than cutaneous melanoma that occur on other body sites. In 2008, a retrospective cohort study by Lachiewicz *et al.* compared the prognosis of patients with scalp/neck melanomas with that of patients with melanomas at other sites in a large, population-based national data set controlling for known prognostic factors. A notable survival difference was found between scalp/neck melanoma and melanoma of other sites even after adjustment for important prognostic factors. The 5- and 10-year Kaplan-Meier survival probabilities for scalp/neck melanoma were 83.1% and 76.2%, respectively, compared with 92.1% and 88.7%, respectively, for melanoma of the other sites, including the extremities, trunk, face, and ears (log-rank test; *P* < 0.001). In a multivariate Cox model, the patients with melanoma of the scalp/neck died of melanoma at 1.84 times the rate of those with melanoma on the extremities (HR, 1.84; 95% confidence interval, 1.62–2.10), when controlling for age, Breslow thickness, sex, and ulceration [5].

Its poor prognosis is probably due to late diagnosis, as the scalp region is often covered by hair and for this reason is not easily accessible to clinical examination. The first clinical symptoms generally appear when melanoma have already reached a significant thickness [6,7,8,9,10]. Scalp melanoma is rare: it represents 2–5% of all skin melanomas and is significantly more frequent in male patients than female. 

It has been observed that scalp melanoma arises within congenital nevi in children and young adults, or within a lentigo maligna in the sun-damaged bald scalp of elderly men. The scalp is also the most common site of desmoplastic melanoma, which has a difficult diagnosis, as it usually lacks the typical clinical features of melanoma.

Dermoscopy is an important tool in the differentiation of pigmented lesions and reduces unnecessary surgery for benign melanocytic lesions. Unfortunately, only few reports have been published regarding dermoscopy of scalp melanoma [11,12] Here, we report the dermoscopic patterns of three patients presenting scalp melanoma. 

## 2. Observations

We report three patients presenting *in situ* scalp melanoma. All patients were male, aged 53, 64 and 71 years old, respectively. All lesions were localized in the sun-exposed bald scalp: two of them in mid-scalp and the other in the vertex. 

**Figure 1:**
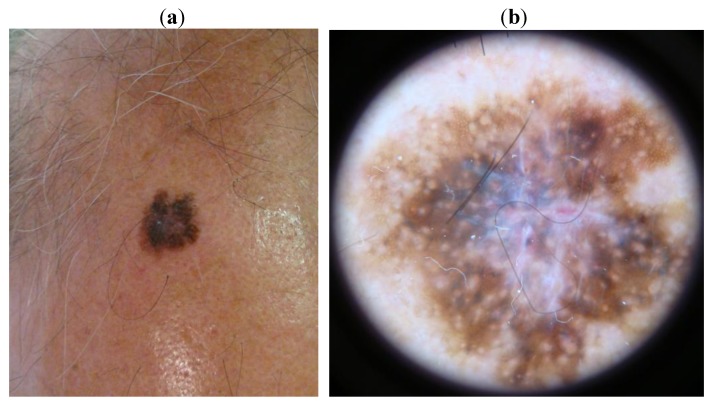
Case 1. (**a**). Naked eye aspect: a 14 mm asymmetric brownish to black patch, with irregular borders; (**b**). Dermatoscopic aspect: a multicomponent pattern, with a dark brown rhomboidal structures around the hair follicles, with peripheral asymmetric, atypical pigment network, and a central blue-grey veil.

The first patient (case 1) presented a 14 mm asymmetric brownish to black patch, with irregular borders. Dermoscopy showed a multicomponent pattern, with dark brown rhomboidal structures around the hair follicles, with peripheral asymmetric, atypical pigment network, and a central blue-grey veil (Figure 1(a,b)). 

**Figure 2:**
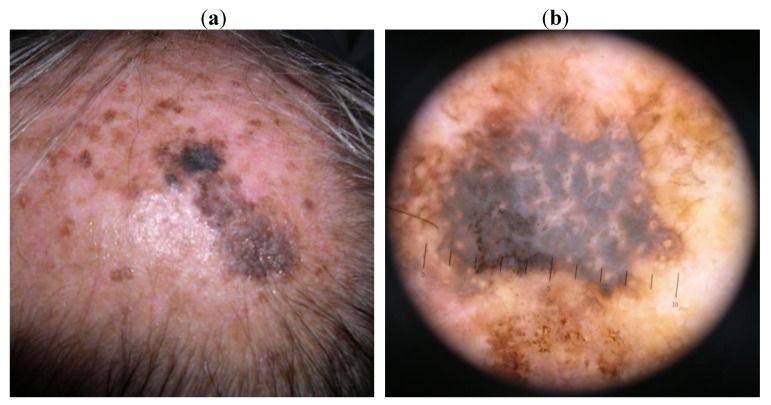
Case 2. (**a**). Naked eye aspect: a 10 mm, irregular black patch among several sebhorreic keratosis; (**b**). Dermatoscopic aspect: a blackish rhomboidal structure, irregular streaks and a central white-blue veil.

The second patient (case 2) presented a 10 mm, irregular black patch among several sebhorreic keratosis, with dermoscopy exhibiting a blackish rhomboidal structure, irregular streaks and a central white-blue veil (Figure 2(a,b)).

**Figure 3:**
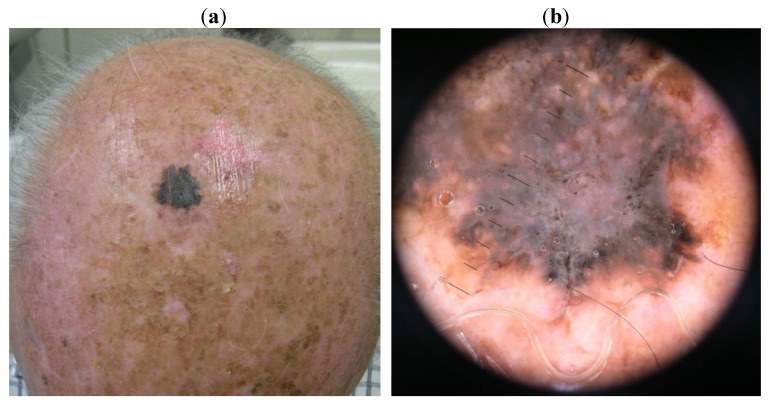
Case 3. (**a**). Naked eye aspect: a 13mm black patch, with irregular borders; (**b**). Dermatoscopic aspect: a black rhomboidal structure, peripheric dots, irregular streaks and a central white-blue veil.

The last patient (case 3) presented a 13 mm black patch with irregular borders, with the dermoscopy showing a black rhomboidal structure, peripheric dots, irregular streaks and a central white-blue veil (Figure 3(a,b)). 

## 3. Discussion

Only few reports had been published about the dermoscopic patterns of scalp melanoma [11,12]. Zalaudek observed that posterior scalp melanomas show the same dermoscopic features typical of trunk/limbs melanomas, such as an atypical pigment network, irregular streaks, and regression structures (pepper-like granules and white scar-like areas); instead, frontal scalp melanomas frequently reveal dermoscopic features of lentigo maligna or lentigo maligna melanoma.

Sahin *et al.* reported a patient who presented clinically with a blue-to-black nodule with satellites, located on the frontal scalp and which clinically resembled melanoma. Dermoscopic examination revealed steel blue botches and bluish perifollicular pigmentation suggestive of the correct diagnosis of blue nevus with satellites, as confirmed histopathologically, showing that for scalp melanoma there are some lesions that can have difficult diagnosis and for this reason it can be useful to improve dermoscopic patterns for the diagnosis [13].

Our cases demonstrate similar dermoscopic patterns observed in three *in situ* melanoma localized in the sun-exposed scalp. Dermoscopy of the three lesions exhibited a lentigo maligna melanoma pattern, presenting irregular rhomboidal structures around the hair follicles. 

More case series of scalp melanoma are needed to establish how reproducible this pattern is on the scalp and the usefulness of dermoscopy in the differential diagnosis of lentigo maligna and lentigo. 

The precise diagnosis of a lentigo maligna is very difficult, even in histologic sections [14]. This implies that even dermoscopy might have some limitations in this set, and thus must be carefully analyzed. However, in our cases dermoscopy was useful, as suspicious dermoscopic findings as irregular rhomboidal structures and white-blue veil were fundamental in establishing the need of performing an immediate biopsy to exclude melanoma.

## 4. Conclusions

Further studies are needed to establish the dermoscopic patterns of scalp melanoma and other pigmented skin lesions localized in the scalp. 

Although scalp melanoma is a rare condition, it is mandatory that dermatologists perform scalp inspections during dermatological examinations, in order to detect lesions early. 
